# Supplementary material for: Knowledge, attitude, and willingness to perform on-site Cardiopulmonary Resuscitation among individuals trained in public CPR: A cross-sectional survey
Source: PLoS One. 2025 May 28;20(5):e0319884. doi: 10.1371/journal.pone.0319884 (PMC12118831; doi:10.1371/journal.pone.0319884)
Supplement: S2 Appendix — (DOCX) [file pone.0319884.s002.docx]

**接受公益CPR培训人员现场实施心肺复苏知识、态度及意愿的调查问卷**

亲爱的朋友们：

您好！非常感谢您于百忙之中填写这份问卷！ 心肺复苏术（以下简称 CPR）是抢救心跳呼吸骤停及保护人脑功能的核心技术。 现实情况中，能否在心搏骤停后最初的关键4min-6min 内开始施行救助，取决于离伤者最近的目击者的行动。本研究目的在于了解接受公益CPR培训人员对心肺复苏的知识掌握程度以及对现场实施 CPR 救援的态度和意愿的影响因素，为今后提高目击者 CPR 施救意愿及院前施救率提供参考。因此特请您填写此问卷，您的支持将对本研究有重要帮助！ 问卷纯属学术研究之用，无所谓对错，您可以畅所欲言表达您的观点和看法，我们将对您填写的内容保密。感谢您的热心参与和配合！

**填写说明:**

1.请在您选择的选项后的方框“口”内打“√”，所有题目均为单项选择，只能选择一个选项;

2.划线部分为自行填写内容，请您根据自己的情况如实填写。

**一、一般资料**

1.您的性别： 男□ 女□

2.民族:

3.宗教信仰: 口有__________ 口无

4.您的年龄:

5. 您的学历：

6. 您的职业：

7. 您有无参加实施 CPR 急救的经历： 有□ 无□

8. 您的亲属中有无冠心病等心源性猝死高危患者： 有□ 无□

9.您有没有目睹过心跳呼吸骤停：有□ 无□

10. 过去 12 个月中您参加公益组织的 CPR 学习经历为________次。

**二、对现场实施心肺复苏的知识、态度、意愿情况**

量表1 心肺复苏知识量表

以下是10题判断题，请根据与自己符合的程度，在表格的相应空格处打“√”。

| 项目 | 正确 | 错误 |
| --- | --- | --- |
| 1.心搏骤停伤员的黄金救治时间只有4-6min，第一目击者应立即实施 CPR。 | √ |  |
| 2.对心搏骤停伤员而言，CPR 是最简单有效的急救措施。 | √ |  |
| 3.意识丧失也可能是心跳呼吸骤停的表现。 | √ |  |
| 4.心脏按压深度是5-6cm。 | √ |  |
| 5.心脏按压的频率是大于100次/分. | √ |  |
| 6.成人心脏按压的位置在两乳头连线的中点位置。 | √ |  |
| 7.成人心肺复苏的按压呼吸比是30:2 | √ |  |
| 8.启动急救医疗措施的时间越短，伤员生存率越高。 | √ |  |
| 9.CPR的现场实行可以不评估环境的安全，挽救生命至上。 |  | √ |
| 10.CPR只要单纯按压就好了，不需要口对口人工呼吸。 |  | √ |

量表2 现场实施心肺复苏态度量表

以下是单项选择题，请根据真实情况，在最符合您情况的一项上打“√”

（5强烈同意 4多数同意 3不确定2多数不同意 1强烈不同意）

| 项目 | 5 | 4 | 3 | 2 | 1 |
| --- | --- | --- | --- | --- | --- |
| 1.我认为及时实施CPR可以挽救很多人的生命。 |  |  |  |  |  |
| 2.在面对心搏骤停伤员时，正确实施 CPR 是一件很容易的事情。 |  |  |  |  |  |
| 3.任何情况下，我都会对心搏骤停伤员实施 CPR。 |  |  |  |  |  |
| 4．CPR 是有价值的，因为它能够挽救心搏骤停伤员的生命。 |  |  |  |  |  |
| 5．我愿意积极去学习正确的心肺复苏术以备不时之需。 |  |  |  |  |  |
| 6．对心跳骤停伤员实施心肺复苏术与我的价值观或宗教信仰并不违背。 |  |  |  |  |  |
| 7．如果我第一时间进行施救了，我相信一定会把他救活。 |  |  |  |  |  |
| 8．我不害怕我的施救会给被施救对象带来一定的损伤。 |  |  |  |  |  |
| 9．我不害怕我的施救行为会给我带来一些不好的事情。 |  |  |  |  |  |
| 10．假如我进行现场施救，我和我的家人都会得到尊重。 |  |  |  |  |  |
| 11．我不会觉得我的施救行为会让我承担法律责任。 |  |  |  |  |  |
| 12．我不害怕因为现场施救而被发现罹患某些疾病（传染病）。 |  |  |  |  |  |
| 13．如果我的家人知道我在现场进行施救了，他们会替我自豪的。 |  |  |  |  |  |
| 14.我不会因为周围没有口对口人工呼吸的防护装置而拒绝实施CPR。 |  |  |  |  |  |
| 15.我会很自责，如果我没有及时对身边的心跳呼吸骤停患者进行急救。 |  |  |  |  |  |

量表3 现场实施心肺复苏意愿量表

请勾出下列适合您的选项，并在相应空格处打“√”

1. 您愿意对下列哪些人实施心肺复苏？(可多选）

口家人 口朋友 口同事 口不认识的人

1. 哪些情况会增加您现场实施心肺复苏的意愿？(可多选）

口对急救人员的法律保护

口实施心肺复苏不会导致自身发生意外伤害

口现场有其他旁观者协助共同施救

口现场急救只需胸外按压

口以上都不会增加我的意愿

1. 您实施心肺复苏会考虑谁意见：(可多选）

口自己 口家人 口朋友 口其他

**感谢您完成这份问卷，为了保证资料的完整性，请您检查一下回答有无漏项。**

**再次感谢您对本课题的支持和参与，祝您工作顺利!**
